# A Systematic Review of the Psychological Implications of Genetic Testing: A Comparative Analysis Among Cardiovascular, Neurodegenerative and Cancer Diseases

**DOI:** 10.3389/fgene.2018.00624

**Published:** 2018-12-10

**Authors:** Serena Oliveri, Federica Ferrari, Andrea Manfrinati, Gabriella Pravettoni

**Affiliations:** ^1^Department of Oncology and Hematoncology, Interdisciplinary Research Center on Decision Making Processes, University of Milan, Milan, Italy; ^2^Applied Research Division for Cognitive and Psychological Science, IEO Istituto Europeo di Oncologia, Milan, Italy

**Keywords:** genetic testing, genetic risk, chronic disease, psychological implication, quality of life, health psychology

## Abstract

**Background:** Genetic testing is performed for different purposes, such as identifying carriers, predicting a disease onset in presymptomatic individuals or confirming a diagnosis. However, these tests may have notable psychological effects, such as generating anxiety and depression. These effects may depend on people's perception of risk, severity, and controllability of the disease; and the availability of treatments. To date, there are no reports that analyze these factors specifically, and their role in influencing genetic test users' experience.

**Methods:** We performed a systematic review of the psychological implication of undergoing genetic testing for cardiovascular, neurodegenerative and cancer diseases. Articles were searched on PubMed, Google Scholar, and PsychInfo.

**Results:** 47 studies were included, 9 concerning cardiovascular disease, 18 neurodegenerative disorders, and 20 for cancer disease. According to the reviewed studies, people experience no significant increase in distress and anxiety, or adverse impacts on quality of life, except the Huntington disease, which is characterized by depressive symptoms, suicidal ideations, and hopelessness in gene carriers. People tend to consider genetic tests as valid information to take important preventive decisions. Genetic risk for cardiovascular disease is perceived to be manageable; genetic analysis for some neurodegenerative diseases (e.g., Alzheimer) or cancer (breast cancer in particular) is considered useful because the problem could be addressed in advance with preventive behaviors.

**Conclusions:** Genetic tests should be proposed along with proper psychological support and counseling focused on users' genetic health literacy; perception of risk, beliefs about disease controllability, in order to foster fruitful medical decisions.

## Introduction

Cancer, cardiovascular diseases and dementia are among the main causes of mortality and morbidity in Europe. Since they will have even larger economic implications in the future, policy-makers have increasingly focused their attention on them (Abegunde et al., 2007; Désesquelles et al., 2014; Mackenbach et al., 2014; Suzman et al., 2015). These conditions affect many people worldwide often causing an impairment of the quality of life and psychosocial well-being, thus they require the attention of the scientific community. In 2015 cardiovascular disease death were 17.92 million, with 422.7 million of cases worldwide (Roth et al., 2017). The WHO estimated that 17.7 million people died from cardiovascular disease last year. Alzheimer disease (AD) affects approximately 24 million people globally (Erkkinen et al., 2018) and this number could quadruple by 2050. Currently, dementia is reported to be the leading cause of mortality in England and Wales (Office for National Statistics ONS), and in 2015 the Eurostat (the Directorate-General of the European Commission) reported 213,000 deaths in Europe caused by nervous system diseases including Alzheimer's.

Regarding cancer data, 14.1 million new cancer cases and 8.2 million cancer deaths occurred in 2012 worldwide (Ferlay et al., 2015), which in 2015 grew to 8.8 million (WHO).

These complex conditions usually need long-term treatments and care, involving different health professionals, expensive drugs, access to medical equipment, putting a large burden on society.

The growing diagnoses of many chronic diseases are associated with an aging population, but also with lifestyle choices such as smoking, diet and exercise, and genetic predisposition (WHO and FAO, 2003; World Health Organisation, 2014).

In the last decades, there have been considerable investments in genomics (DNA-based) research to study susceptibility to cancer and other chronic diseases and to promote new preventive interventions (Walter and Emery, 2012). Currently, the use of family health history and multiplex genetic tests to identify an individual's risk for multiple diseases simultaneously is a frequent clinical practice (Yang et al., 2003; Yoon and Scheuner, 2003; Khoury et al., 2004; Yoon, 2005). Many associations between single-nucleotide polymorphisms (SNPs) and risks for common complex diseases have been identified. Genetic testing generally provides information about the presence of these genetic variants (SNPs), which could represent an increased risk of developing the disease. Their clinical utility depends on how much the knowledge about this genetic variant could give additional information concerning diagnosis, prognosis or contribute to disease management. Available types of testing include for instance diagnostic, carrier, predictive and susceptibility tests. Diagnostic tests confirm a diagnosis when a particular condition is suspected, based on physical symptoms. Carrier testing identifies people who carry one copy of a gene mutation that can be inherited by their offspring. Predictive and susceptibility testing identifies mutations that increase a person's risk of developing disorders with a genetic basis. These tests may help people making decisions about their daily habits or medical care. For instance, discovering the susceptibility for breast cancer, or stroke could allow people to change their lifestyle, nutrition and “take steps to reduce those risks for which interventions are or will be available” (Collins and McKusick, 2001). Nonetheless, not all kinds of genetic testing are useful for clinical management or outcomes improvement, either because of a lack of treatments, uselessness for the personal decision, or absence of scientific evidence for the genetic predisposition. Proven clinical utility and cost-effectiveness need to be carefully evaluated when considering the implementation of genetic testing in healthcare (Cornel et al., 2014), even when considering the recent discoveries which have underlined the heterogeneity of chronic diseases and the importance of gene-environment interaction in modulating disease onset and responses to preventive interventions (Curtis et al., 2012).

In spite of these premises, a recent study, published by the Market Research Future (the “Global Genetic Testing Market - Forecasts from 2018 to 2023” see https://www.marketresearchfuture.com/), reported the amount of genetic tests performed in the European and non-European countries based on the subdivision of pathologies or on the type of genetic test (diagnostic, predictive, etc.). The study estimated a global growth of genetic testing market at a Compounded Average Growth Rate (CAGR) of 12.94% by 2023. This exponential growth should go hand in hand with an appropriate genetic counseling practice, but to date genetic information is often given to people with poor genetic literacy without a specific psychological assessment (Burke et al., 2002) or genetic testing users do not receive a specific pre and post-test counseling (Janssens et al., 2017). For instance, in Italy, only 12% of all genetic analyses had been accompanied by pre or post-test counseling (Giardino et al., 2016).

The genetic counseling is usually provided by trained professionals, mainly geneticists, who explain the genetic aspects of illnesses and the risk of developing or passing an illness to their offspring (WHO). It should address patients' concerns, and one of their families, to help with the decision-making process. Nevertheless, this not always happened in recent years especially with the introduction of direct to consumers genetic testing (DTC), genetic tests sold to people without a medical intermediate (Oliveri and Pravettoni, 2016).

Starting from these premises, we could infer that genetic risk communication might deeply affect people's lives and habits. To date, studies on the psychological impact of genetic tests mainly focused on “harmful” reactions, such as anxiety, distress, and depression, when receiving genetic risk information, obtaining discordant results and without any explanation/discussion for this discrepancy (Oliveri et al., 2016a). Previous reviews revealed that DNA based disease risk has little or no effect on health-related behaviors (Heshka et al., 2008; Hollands et al., 2016). We should consider that genetic testing impact also depends on how people perceive their risk, severity, and controllability related to specific categories of disease (Cameron and Muller, 2009; Wang et al., 2009; Wade et al., 2012); on the genetic tests predictability or nature of the diseases (from monogenic to genetic susceptibility factors), and on the presence/absence of treatments (Cameron and Muller, 2009).

People's emotional reactions to genetic testing and how risk perceptions vary from diseases to disease are fundamental aspects to be investigated on, in order to correlate preventive behaviors they may, or may not, adopt (DiLorenzo et al., 2006; Shiloh et al., 2013).

For this reason, we aim to provide a comprehensive overview of studies realized in the last two decades (2000–2016), which investigated psychological and behavioral issues after having undergone genetic testing for different categories of chronic diseases. The purpose is to identify a limited number of overarching psychological reactions for each condition. In particular, we chose to compare neurodegenerative, cardiovascular and cancer diseases, because of their differences in treatments availability and preventive options (e.g., there are fewer preventive options for neurodegenerative disorders compared to cancer or cardiovascular diseases, where risk is in some cases manageable with screenings or healthier lifestyles). Moreover, people have different beliefs and perception of the controllability for these diseases which could affect their reaction to a positive genetic test result.

## Methods

### Study Design and Search Strategy

Potential eligible articles were systematically searched on PubMed, Google Scholar and PsychInfo using the following combinations of terms: “psychological outcomes,” “psychological impact,” “genetic test,” “genetic risk,” “neurodegenerative disorders,” “cancer,” and “cardiovascular disease.” Depending on the disease for which the genetic test was performed, we allocated the collected articles into three general categories: Cancer (C), Cardiovascular diseases (CV), and Neurodegenerative disorders (N) (see Table 1). Following criteria were considered to include articles:

- studies in which a psycho-behavioral and/or quality of life evaluation after having received genetic test results was performed;
- studies in which subjects tested were adults.

**Table 1:** Characteristics of studies evaluating psychological impact of genetic testing for cardiovascular, neurodegenerative and cancer diseases.

	**Dc**	**Author**	**Year/Country**	**Population**	**Subject of evaluation (disease specific)**	**Design**	**Used instruments behavioral changes, coping, QoL, wellbeing and symptoms**	**Used instruments psychological impact**	**Main findings**
**CARDIOVASCULAR DISEASES**									
1	CV	Hickey et al. (a)	2014 (USA)	♀♂adults (*n* = 58) with cardiac genetic diagnoses	Cardiac genetic testing	After testing	SF-36	HADS-A, HADS-D, IPQ-R	Positive genetic results did not negatively impact patient well-being with the exception of the bodily pain domain of the SF-36
2	CV	Hickey et al. (b)	2014 (USA)	♀♂adults (*n* = 31) ***gc, nc***	Cardiac genetic testing	After testing	SF-36		Physical components of the SF-36 were within normal limits (46.2 ± 6.6) but elevated for mental components (59.9 ± 5.3)
3	CV	Christiaans et al.	2009 (GERMANY)	♀♂adults (*n* = 228) MYBPC3, MYH7, TPM1, TNNT2, TNNI3, PRKAG2 and GLA ***gc*** with and without symptoms	Hypertrophic cardiomyopathy y (HCM)	After testing	SF-36; IPQ-R Perceived risks 10-point response options (“very small” to “very large”)	HADS-A, HADS-D	QoL and distress were worst in ***gc*** with manifest HCM before DNA testing and best in predictively tested ***gc*** without HCM Illness and risk perception related variables were major determinants of QoL and distress
4	CV	Jones & Clayton	2012 (USA)	♀♂adults (*n* = 70) affected or at-risk BMPR2 mutations	Primary pulmonary arterial hypertension	Before/After testing		IES	Test acceptors evidenced dramatic changes in levels of distress, often meeting levels associated with PTSD prior to testing, and evidencing dramatically lower levels of distress following testing Not observed differences in levels of distress between ***gc*** and ***nc***
5	CV	van Maarle et al.	2001 (NETHERLANDS)	♀♂adults(*n* = 677) ***gc**, **nc***	(FH) Familial hypercholesterolaemia	Before/After testing	SF-36, EuroQol	HADS	Effects on mood were minimal to absent, as were general QoL effects
6	CV	Marteau et al.	2004 (UK)	♀♂adults (*n* = 341) at risk and adult relatives (*n* = 128)	(FH) Familial hypercholesterolaemia	Before/After testing	Items from RIPQ; Self-reports about smoking, diet, activity level and cholesterol-lowering medication adherence	STAI short form, HADS	Finding a mutation had no impact on perceived control or adherence to risk-reducing behavior ***gc*** believed less strongly in the efficacy of diet in reducing their cholesterol level and showed a trend in believing more strongly in the efficacy of cholesterol-lowering medication
7	CV	Hietaranta-Luoma et al.	2015 (FINLAND)	♀♂adults and young adults (*n* = 107) high-risk vs. low-risk group vs. control group (*n* = 56)	APOE susceptibility testing for Cardio-vascular disease	Before/After testing	*Ad hoc* questionnaire (Diet, alcohol consumption), HTAS	STAI	Psychological effects of personal genetic risk information were shown to be short-term Slightly higher levels of state anxiety and threat experienced in the high-risk group compared to baseline Information on the ApoE genotype impacted the experience of cardiovascular threat; this effect was most intense immediately after genetic feedback was received
8	CV	Hendriks et al.	2008 (NETHERLANDS)	♀♂adults (*n* = 134), relatives of LQTS patients, with abnormal, uncertain, or normal ECG	Long qt syndrome (LQTS)	Before/After testing		IES, BDI	Individuals with an abnormal ECG (i.e., clinical diagnosis of LQTS) expressed a moderate level of anxiety that was not affected by mutation carrier status Individuals with a normal or uncertain ECG likewise displayed a moderate level of anxiety at baseline Individuals with an initial uncertain ECG who were later identified as genotype-positive maintained moderate disease-related anxiety over time, although depression scores declined to a level comparable to the general population
9	CV	Legnani et al.	2006 (ITALY)	♀♂ adults and relatives (n = 140) ***gc**, **nc, control group***	Thrombophilia alterations	Before/After testing	Perceived health status, perceived well-being, and perceived daily-life stress	CBA-H anxiety, health fears, depressive reactions, moods	For both groups ***gc*** and ***nc***, none of the psychological variable scores showed significant worsening, at pre and post-test Anxiety significantly decreased at post-test in adults with thrombophilia alterations Diagnosis of thrombophilia alterations seemed to be well accepted in the short term
**NEURODEGENERATIVE DISORDERS**									
10	N	Decruyenaere et al.	2003 (BELGIUM)	♀♂adults (*n* = 57) ***gc*** and ***nc***	HD	Before/After testing		STAI, BDI, SCL-90, IES, HOS, MMPI	5 years after the test, mean distress scores of both ***gc*** and ***nc*** were within the normal range ***gc*** had significantly less positive feelings and were more consciously avoiding HD-related situations and thoughts (Avoidance) Compared with baseline level, mean depression, general and specific anxiety had significantly decreased 1 year and 5 years post-test Persons who asked the test to get rid of the uncertainty, without being able to specify implications for substantial life areas, had more psychological distress before and after the test than those who wanted the test for specific reasons
11	N	Licklederer et al.	2008 (GERMANY)	♀♂ adults (*n* = 121) ***gc**, **nc***, and patients with HD	HD	After testing	SF-12, German Social Support Questionnaire, SOC-L9, BFS	BDI-II, BSI, GSI	Comparable mental health and QoL in ***nc*** and ***gc*** without symptoms of HD Patients with manifest HD showed a higher level of depression and lower QoL than ***nc*** and ***gc*** without symptoms In ***nc*** increased depression and low mental QoL were due to: low perceived social support, no intimate relationship, female sex and younger age For ***gc*** increased depression and low mental QoL were due to: low perceived social support, the expectation of an unfavorable genetic test result before the testing procedure and being childless
12	N	Almqvist et al.	2003 (CANADA)	♀♂ adults (*n* = 106) ***gc**, **nc***	HD	Before/After testing	GWS	BDI, SCL-90-R	Adverse events, e.g., suicide, clinical depression occurring in a part of both groups but more frequently in ***gc*** Overall improvement in psychological distress compared to baseline in both groups More depressive symptoms in ***gc*** than in ***nc*** 5 years after testing Adverse events, e.g., suicide, clinical depression occurring in a part of both groups but more frequently in ***gc*** Overall improvement in psychological distress compared to baseline in both groups More depressive symptoms in ***gc*** than in ***nc*** 5 years after testing Adverse events, e.g., suicide, clinical depression occurring in a part of both groups but more frequently in ***gc*** Overall improvement in psychological distress compared to baseline in both groups More depressive symptoms in ***gc*** than in ***nc*** 5 years after testing -Adverse events, e.g., suicide, clinical depression occurred in both groups ***gc*** and ***nc***, but more frequently in ***gc***-Overall improvements in psychological distress compared to baseline in both groups were registered -More depressive symptoms in ***gc*** than in ***nc*** 5 years after testing
13	N	Timman et al.	2004 (NETHERLANDS)	♀♂ adults (n = 142) ***gc**, **nc***	HD	Before/After testing	GHQ-28	BHS, IES	Long-term increase in hopelessness in ***gc*** compared to baseline No long-term improvement in hopelessness in ***nc*** compared to baseline Improvement in long-term in avoidance and intrusions in both groups compared to baseline but more pronounced for ***nc***
14	N	Larsson et al.	2006 (SWEDEN)	♀♂ adults (*n* = 93) ***gc**, **nc***	HD	Before/After testing	Well-being: 150-millimeter- long line on a visual analog scale. GHQ-30, SIBS, LSI, LSA	BDI	Both ***gc*** and ***nc*** showed high suicidal ideation before the predictive testing Depression scores and frequency of suicidal thoughts increased in ***gc*** over time No differences regarding life satisfaction and life style
15	N	Horowitz et al.	2001 (USA)	♀♂ adults (*n* = 59) ***nc, gc*** with and without HD neurological symptoms	HD	Before/After testing		IES, BDI	***gc*** with neurological impairment had significantly higher psychological symptom scores at baseline than ***gc*** without neurological impairment or ***nc*** For ***nc*** and ***gc*** without HD neurological symptoms the news of genetic testing for the HD gene had limited detrimental impact
16	N	Gargiulo et al.	2009 (FRANCE)	♀♂ adults (*n* = 119) ***gc**, **nc***	HD	After testing		BDI,BHS, STAI, IES	Depression was frequent in asymptomatic ***gc*** (58%) 27% of ***nc*** did not cope well with a favorable result, and a significant percentage of ***nc*** (24%) were depressed during follow-up A previous episode of depression was predictive of depression after genetic testing in both ***gc*** and ***nc***
17	N	Robins Wahlin et al.	2000 (SWEDEN)	♀♂ adults (*n* = 34) ***gc**, **nc***	HD	Before/After testing	GHQ-30, SIBS, Risk perception (VAS); LSI, LSA	BDI	Both groups showed high suicidal ideation and self-injurious behavior ***nc*** had a very high frequency of attempted suicide, and both groups had similarly pronounced psychiatric dysfunction
18	N	Witjes-Ané et al.	2002 (NETHERLANDS)	♀♂ adults (*n* = 134) ***gc**, **nc***	HD	Before/After testing	UHDRS		***gc*** complained more than ***nc*** about sadness, low self-esteem, aggressive behavior, and compulsions At 18-months follow-up ***gc*** still complained about aggression, while complaints about mood and low self-esteem had disappeared
19	N	Surampalli et al.	2015 (USA)	♀♂adults (*n* = 29) ***gc**, **nc***	VCP gene for Hereditary myopathies (HIBM), Paget's disease of bone, Fronto-temporal dementia	Before/After testing	Risk Perception and Symptom Specific Concern (five point Likert scale), RBD	HADS	At baseline, one quarter of the participants had high levels of anxiety and nobody was depressed Scores were in normal range one year following testing Mean risk perception at baseline was 50.1%
20	N	Gonzalez et al.	2012 (ISLAND OF FLORES AND S. MIGUEL)	♀♂adults (*n* = 47) ***gc**, **nc***	Pre-symptomatic testing for Machado-Joseph disease (MJD)	After testing	PGWB		Scores indicating moderate or severe stress were found in half of participants The average score in the PGWB scale was lower in symptomatic than in asymptomatic subjects Impact of the appearance of first symptoms on the psychological state
21	N	Gooding et al.	2006 (USA)	♀♂adults (*n* = 60) and parents affected with AD	APOE susceptibility testing for AD	After testing	Interview -perceived likelihood of developing AD; -beliefs about the causes of AD; perceived control over AD;	Interview -reactions to receiving results	Most participants viewed genetic testing as providing valuable information that could help direct future health care decisions, to seek information about health threats, and need to feel in control of their health
22	N	Linnenbringer et al.	2010 (USA)	♀♂adults (n = 246) and parents affected with AD	APOE susceptibility testing for AD.	Before/After testing	Perceived personal risk to develop AD: a scale of 0–100%. Understanding of risk results, AD concern, severity of AD, AD treatment optimism, personal control (5-point Likert scales)	BAI, CESD	69.3% of participants, who accurately recalled their AD risk assessment 6 weeks after risk disclosure, believed that their AD risk was higher than risk estimate they were given This group of people had higher scores in AD control and anxiety at baseline Overall, anxiety scores were below clinically significant levels
23	N	Cassidy et al.	2008 (USA)	♀♂ adults (*n* = 123) APOE vs. dominant mutations carriers	APOE susceptibility testing vs. autosomal dominant mutations (presenilin-1, presenilin-2, or TAU genotype) for AD or frontotemporal dementia	After testing		IES	The test-related distress experienced by those receiving positive results for a deterministic mutation was similar to the distress experienced by those receiving positive results from genetic susceptibility testing The majority of participants receiving genotype disclosure did not experience clinically significant distress 1 year after learning of their test results
24	N	Green et al.	2009 (USA)	♀♂asymptomatic adults (*n* = 162) and parents with AD REVEAL STUDY	APOE for AD, Disclosure group vs. non-disclosure group	Before/After testing		BAI, CESD, IES	The disclosure of APOE genotyping results to adult children of patients with Alzheimer's disease did not result in significant short-term psychological risks Test-related distress was reduced among those who learnt that they were APOE ε4–negative Persons with high levels of emotional distress before undergoing genetic testing were more likely to have emotional difficulties after disclosure
25	N	Chao et al.	2008 (USA)	♀♂ asymptomatic adults (*n* = 162) and parents with AD REVEAL STUDY	APOE for AD, Disclosure group vs. non-disclosure group	After testing	Yes/no questions about changes in: Any behavior specific to AD prevention Medications/vitamins Diet Exercise		12 months after disclosure, APOE-positive participants reported changes in any one of the domains of diet, physical exercise and medication or vitamin intake, more often (52%) than ε4-negative participants (24%) or the nondisclosure group (30%) Within each domain, there were no significant differences between the groups (disclosure vs. non-disclosure group)
26	N	Vernarelli et al.	2010 (USA)	♀♂ adults (*n* = 272) REVEAL STUDY	APOE for AD	After testing	Yes/no questions with free-text field on changes in: Overall diet use of dietary supplements exercise		Genetic susceptibility testing for AD was positively associated with dietary supplement use after risk disclosure. Such changes occurred despite the absence of evidence that supplement use reduces the risk of AD
27	N	Romero et al.	2005 (USA)	♀♂ adults (*n* = 76) ***gc**, **nc***	APOE	After testing		Self-developed questionnaire	***nc*** did not feel worried or depressed, but they felt relieved 15–30% of ***gc*** felt depressed and 11–22% felt worried. A little percentage also felt relieved
**CANCER**									
28	C	Aspinwall et al.	2013 (USA)	♀♂ adults (*n* = 60) unaffected ***nc*** (*n* = 27),unaffected ***gc*** (*n* = 15), affected ***gc*** (*n* = 18)	CDKN2A/p16 mutations risk for melanoma and pancreatic cancer	Before/After testing	Open-ended questions on costs and benefits of Genetic Testing	HADS, MICRA, 3-item melanoma and pancreatic cancer worry	Low reported anxiety and depression For ***gc*** and ***nc***, anxiety decreased significantly throughout the 2-year period Depression and melanoma worry showed short-term decreases Worry about pancreatic cancer was low and decreased significantly In all groups, test-related distress and uncertainty (MICRA) were low, regret was absent, and positive experiences were high All participants reported at least one perceived benefit of genetic testing
									***gc*** reported increased knowledge about melanoma risk and prevention and increased prevention and screening behaviors for self and family ***nc*** reported increased knowledge and emotional benefits
29	C	Lammens et al.	2010 (NETHERLANDS)	♀♂ adults (*n* = 119) ***gc**, **nc***	p53 germline mutation Li-Fraumeni Syndrome (LFS)	After testing	SF36, Questionnaires perceived risk, social support, motivations to undergo or not undergo genetic testing and regrets	IES, Social Constraint Questionnaire, CWS	Uptake of pre-symptomatic testing was 55% 23% reported clinically relevant levels of LFS-related distress. ***- gc*** were not significantly more distressed than ***nc*** or than those with a 50% risk who did not undergo genetic testing Those with a lack of social support were more prone to report clinically relevant levels of distress
30	C	Di Prospero et al.	2001 (CANANDA)	♀ adults (*n* = 24) unaffected	BRCA 1/2 *B/Ov*	After testing	*Ad Hoc* questionnaire: Communication with family members, attitudes toward surveillance, prevention options, satisfaction with clinical services, need for additional support, and satisfaction with decision to undergo genetic testing	Focus group and *ad hoc* questionnaire for perception of cancer risk and worry assessment	Cancer risk perception and worry increased after receipt of the test results Participants did not regret their decision to undergo testing Confidence in the efficacy of cancer surveillance was high Prophylactic oophorectomy was much more acceptable than prophylactic mastectomy 38% of the participants felt they would benefit from a support group
31	C	Ertmanski et al.	2009 (POLAND)	♀ adults (*n* = 3,524) affected and unaffected	BRCA1/2 *B/Ov*	Before/After testing	Patient satisfaction	STAI, BHI-12, IES	Anxiety does not increase in women positive for BRCA1 Women who experience high levels of anxiety before genetic testing continue to experience high levels of anxiety up to 1 year post testing BRCA1 ***gc*** with a previous diagnosis of cancer had significantly higher levels of cancer-related distress at 1 month post-test than those without cancer
32	C	Hamilton et al.	2009 (USA and CANADA)	♀ adults (*n* = 7) non symptomatic individuals	BRCA1/2 *B/Ov*	After testing	Follow-up interviews Impact on daily life, health behaviors in the intervening years.		Participants accepted recommended surveillance and preventative measures to maximize a healthy lifestyle and reported both the benefits of knowing their mutation status as well as challenges they had encountered since testing Over time, awareness of genetic risk does not appear to diminish
33	C	Vos et al.	2012 (NETHERLANDS)	♀ adults (*n* = 248) ***gc***, unclassified-variants and uninformative-results	BRCA1/2 *B/Ov*	After testing	IPQ-R, COPE *Ad hoc* questionnaire: perception of risk, medical outcomes, familial and psychological contexts	RSPWB	The actually communicated cancer risks did not directly predict any outcomes (lifestyle changes, medical intentions and emotions) The counselees' perception of risk and heredity likelihood predicted medical intentions, behaviors, physical and psychological life-changes, stigma, mastery, negativity and cancer-worries Short-term distress was related to the perception not only of their own risks, but also of their relatives' risks and heredity-likelihood
34	C	Katapodi et al.	2011 (USA)	♀ adults (*n* = 372) affected probands and relatives not tested	BRCA1/2 *B/Ov*	After testing	IPQ-R, DCS *Ad hoc* questionnaire Perceived risk (scale 0-100), knowledge risk factors and modes of gene inheritance, family relationships, family communication	Psychological distress (scale 0–10)	Probands perceived higher risk and had more psychological distress associated with breast cancer Probands had more knowledge regarding risk factors and gene inheritance, and greater decisional conflict regarding genetic testing Relatives reported higher perceived severity and controllability No differences were observed in family relationships and family communication between probands and relatives
35	C	Lodder et al.	2000 (NETHERLANDS)	♀ adults (*n* = 78) healthy subjects, ***nc**, **gc***	BRCA1/2 *B/Ov*	Before/After testing	Interview transcripts about intentions concerning risk management, reported impact of the test outcome	HADS, IES	High post-test anxiety was reported by 20% of the ***gc*** women and by 35% of their partners 11% of women ***nc*** and 13% of their partners reported high post-test anxiety levels ***nc*** who had a sister identified as a ***gc*** had higher post-test levels of depression than the other ***nc***
36	C	Meiser et al.	2002 (AUSTRALIA)	♀ adults (*n* = 143) ***nc**, **gc*** and not tested	BRCA1/2 *B/Ov*	Before/After testing	MBSS, satisfaction with the decision to undergo testing	BAI, STAI-S, IES	Compared with women not offered testing, ***gc*** had significantly higher distress 7–10 days and 12 months post results ***nc*** showed a significant decrease in state anxiety 7–10 days post-notification and in depression 4 months post-notification compared with women not offered testing
37	C	Rini et al.	2009 (USA)	♀ adults (*n* = 182) affected probands	BRCA1/2 *B/Ov*	After testing	*Ad hoc* questionnaire (Likert scale): decision status, perceived risk for relapse benefits/barriers to mammography benefits of and barriers to risk-reducing mastectomy	MICRA, BSI, IES, DCS	Substantial numbers of women reported elevated decisional conflicts 1-month and 12-months post disclosure, health beliefs and emotional factors predicted decisional conflict at different time points, with health beliefs more important 1 month after test disclosure and emotional factors more important 1 year later
38	C	Smith et al.	2008 (USA)	♀ adults (*n* = 126) ***gc*** and women elected not to be tested	BRCA1/2 *B/Ov*	Before/After testing	Perceived risk, SF36	SCL-90-R, GSI, IES, PSC, CESD, STAI-Form Y1	Results indicated no systematic effects of testing based on personal cancer history ***gc*** and women elected not to be tested reported greater perceived risk and intrusive and avoidant thoughts at follow-up time points than did women who received negative (uninformative) or variant results ***gc*** reported more distress at the 3-month follow-up but by 6 months the effects of test result on distress dissipated and groups were comparable
39	C	Dougall et al.	2009 (USA)	♀ adults (*n* = 126)	BRCA1/2 *B/Ov*	Before/After testing	COPE	SCL-90-Revised, IES, PSC, STAI CESD	Coping was relatively stable over time and did not vary as a function of genetic test results Active coping strategies were used more often by women with a personal cancer history than by women without cancer Use of avoidant coping was reliably and positively associated with distress over time, independently of cancer history and test result
40	C	Samson et al.	2014 (CANADA)	♀ adults (*n* = 6) with family history of ***B/Ov***	BRCA1/2 *B/Ov*	After testing		Grounded theory interview	Main topics: Physical Task: attempting to limit the impact of the test result; Psychological Task: living with uncertainty; Social Task: finding effective support
41	C	Arver et al.	2004 (CANADA)	♀ adults (*n* = 87) healthy women, ***gc**, **nc***	BRCA1, BRCA2, MLH1, and MSH2 *B/Ov* and colon cancer susceptibility genes	Before/After testing	SF-36, satisfaction with decision making and testing (5-graded scale)	HADS	Significant decrease in anxiety scores over time The levels of depression in ***gc*** decreased over time while, surprisingly the levels in ***nc*** increased, however, still within the normative range Vitality was the only domain that was statistically significantly influenced by gene testing. In ***gc*** of colon cancer genes the scores dropped two to 6 months after the result disclosure, followed by increased levels, while the levels in ***nc*** were more stable
42	C	Claes et al.	2004 (SWEDEN)	♀ adults (*n* = 62) ***gc**, **nc***, and patients with an inconclusive (no specific familial mutation is found) genetic test result	BRCA1/2 *B/Ov*	After testing	Semi-structured interviews about personal experiences with cancer, family history of ***B/Ov*** cancer, genetic testing. Questions on willingness for testing, perceived seriousness and perceived control	UCL, STAI, SCL-90, IES	***Gc*** felt more in control, but they also reported negative emotional impact and being concerned about their children ***Nc*** were relieved No differences among groups in general and cancer-specific distress No differences among groups regarding perceived seriousness of breast and ovarian cancer and perceived control of breast cancer Perceived control of ovarian cancer was highest in the inconclusive group
43	C	Manchanda et al.	2015 (UK)	♀♂ adults (*n* = 1,034) family history based testing (FH) vs. population-based genetic testing (PS)	BRCA1/2 *B/Ov*	After testing	SF-12	HADS, HAI, MICRA	There were no statistically significant differences on anxiety, depression, distress, uncertainty and quality-of-life between the FH and PS arms, at 7 days or 3 months after genetic testing. Overall anxiety and uncertainty associated with genetic testing decreased Positive experience scores increased
44	C	Shiloh et al.	2013 (ISRAEL)	♂ adults (*n* = 81) ***gc**, **nc***	BRCA1/2 *B/Ov*	After testing	Brief IPQ, Questions about health behavior, questions about risk perception	MICRA	Up to 4 years post genetic testing, 48% of those who tested positively reported that the test increased their perceptions of risk, and 74% of them increased surveillance for cancer ***nc*** did not report increased perceived risk and relatively few increased surveillance (31%) ***gc*** were significantly more distressed from testing
45	C	Claes et al.	2005 (BELGIUM)	♀ adults (*n* = 68) unaffected ***gc**, **nc***	*B/Ov*	Before/After testing	UCL, Questions about Perceived impact of genetic test, Questions about illness representation, SCL-90	IES, STAI	Mean levels of distress were within normal ranges in both ***gc*** and ***nc*** Cancer-specific distress and state-anxiety significantly decreased in ***nc*** from pre to post- test while general distress remained about the same There were no significant changes in distress in the group of ***gc*** except for ovarian cancer distress which significantly decreased from pre- to post test The study did not reveal adverse effects of predictive testing when offered in the context of a multidisciplinary approach
46	C	Andrews et al.	2004 (UK)	♀ adults (*n* = 43) ***gc**, **nc***	BRCA1/2 *B/Ov*	Before/After testing	*Ad hoc* questionnaire: satisfaction with genetic testing perceived Risk of developing breast cancer accuracy of perceived risk	IES, STAI, BDI	Women who chose to learn their results had significantly higher baseline breast cancer anxiety, compared to those who choose not to learn their results Unaffected women who choose to learn their results showed a significant decrease in breast cancer anxiety 4 months and 12 months post-notification compared to baseline Genetic testing does not lead to adverse psychological outcomes
47	C	Reichelt et al.	2004 (NORWAY)	♀ adults (*n* = 395) with or without personal history of disease	BRCA1/2 *B/Ov*	Before/After testing	GHQ-28	HADS, IES, BHS	No significant changes were found in psychological distress from baseline to follow-up in any groups Women with cancer were significantly more distressed at baseline and at 6 weeks

***Dc**, Disease category; **CV**, Cardiovascular disease and other vascular pathologies; **C**, Cancer; **N**, Neurological disorders*.

Used abbreviations:

***♀**, female; **♂**, male; **AD**, Alzheimer disease; **HD**, Huntington disease; **B/Ov**, breast and/or ovarian cancer; **QoL**, Quality of life; **gc**, gene carrier/mutation carriers; **nc**, non-carriers*.

Abbreviations of used instruments:

*BAI, Beck Anxiety Inventory; BDI, Beck's Depression Inventory; BFS, Benefit Finding Scale; BHI-12, Basic Hope Inventory. BHS, Becks Hopelessness Scale; BSI, Brief Symptom Inventory; CBA-H, Cognitive Behavioral Assessment Hospital Form; CESD, Center for Epidemiologic Studies Depression Scale; COPE, inventory of coping responses; CWS, Cancer Worry Scale; DCS, Decisional Conflict Scale; GHQ-28- GHQ-30, General health questionnaire 28-items/30-items;GSI, Global Severity Index; GWS, General Wellbeing Scale; HADS, Hospital Anxiety and Depression Scale; HAI, Health Anxiety Inventory; HOS, Health Orientation Scale; HTAS, Health and taste attitude; IES, Impact of Event Scale; IPQ-R, Illness Perception Questionnaire – Revised; LSA, Life-Styles Assessment; LSI, Life Satisfaction Index; MBSS, Miller Behavioral Style Scale; MICRA, The Multidimensional Impact of Cancer Risk Assessment; MMPI, Minnesota Multiphasic Personality Inventory; PGWB, Psychological General Well-Being Index; PSC, Perceived Stress Scale; RBD, Risk Behavior Diagnostic Scale; RIPQ, Revised Illness Perceptions Questionnaire; RSPWB, The Ryff Scales of Psychological Well-Being; SCL-90, The Symptom Checklist; SF-36/SF-12, Short-Form Health Questionnaire 36-Item/12-Item; SIBS, Self-Injurious Behavior Scale; SOC-L9, Leipzig Sense of Coherence Scale; STAI, State Trait Anxiety Inventory; UCL, Utrecht Coping List; UHDRS, Unified Huntington′s Disease Rating Scale*.

Exclusion criteria were determined by the aim to analyze the “raw” impact genetic test information can have on people's psychological reactions and/or quality of life, without the mediation of interventions (e.g., counseling), or specific population (e.g., children) or phenomenon (e.g., Direct to Consumer genetic testing).

Exclusion criteria were as follow:

- investigation of prenatal screening, or childhood and adolescent genetic testing;
- investigation of genetic testing for psychiatric disorders;
- investigation of family dynamics, the efficacy of psychological or other kind of educational and counseling intervention;
- hypothetical situations in undergoing genetic testing;
- direct to consumer genetic testing.

Recent reviews, meta-analyses or narrative accounts of knowledge were excluded.

For each study, we identified the implemented design, the number, and composition of participants, the psychological instruments employed and the main findings regarding psycho-behavioral outcomes and quality of life after testing.

Because of substantial heterogeneity among research studies, no attempt at formal meta-analysis was made in this contribution.

## Results

### Studies Selection and Characteristics

Three thousand and three hundred twenty-eight manuscripts, published between 2000 and 2016, were assessed for eligibility by reading title and abstract. In total 90 studies were potentially eligible. After reading the full text, articles were included for qualitative synthesis only if they met inclusion/exclusion criteria. As a consequence, 43 studies could not be included, mostly because they investigated prenatal screening or childhood diseases, psychiatric disorders, family dynamics, the efficacy of the psychological intervention, genetic counseling effects or hypothetical situations in undergoing genetic testing. We considered these studies suitable to be treated as a separate topic.

**Figure 1:**
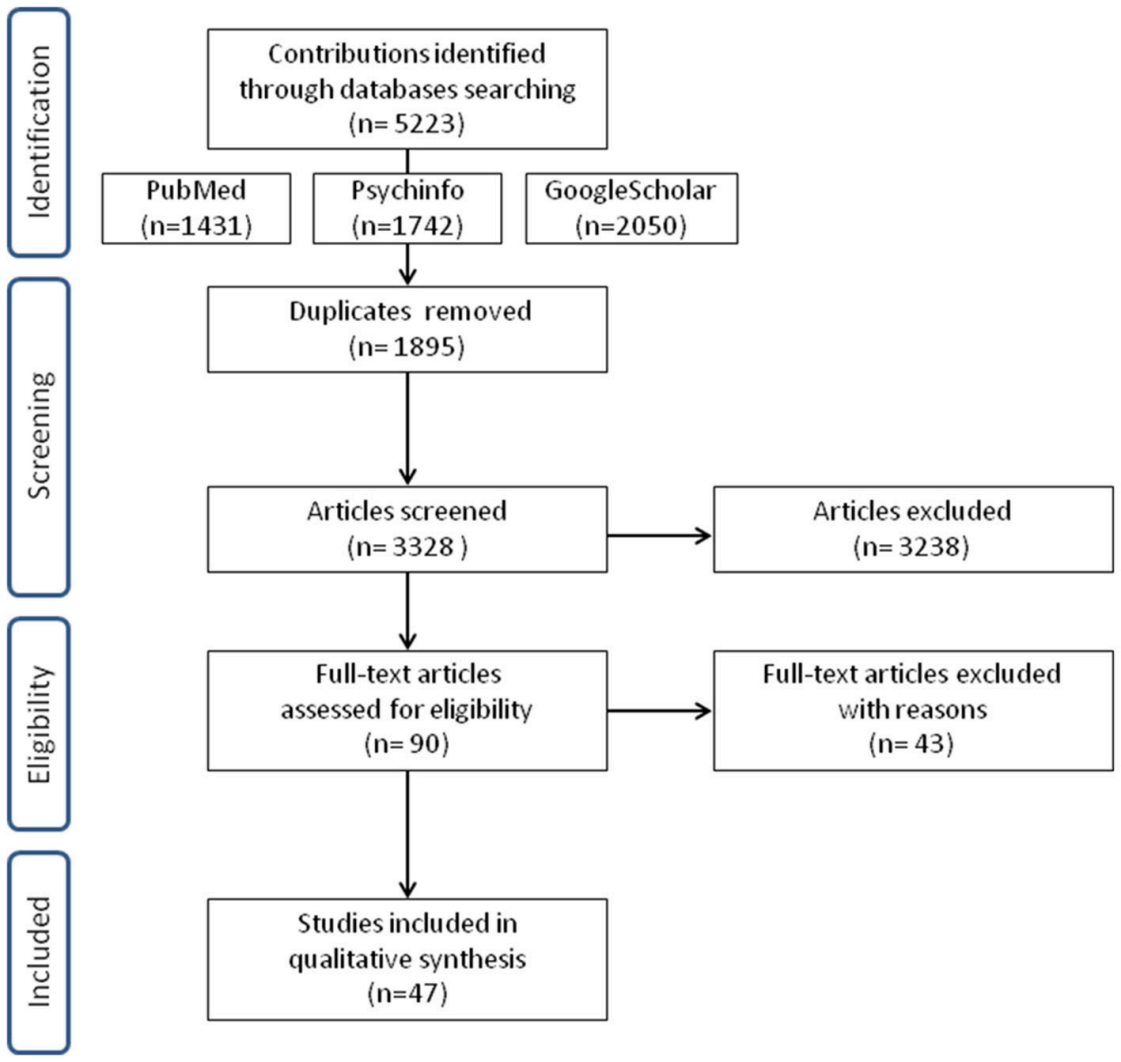
Flow diagram describing the study selection.

Finally, a set of 47 studies met our inclusion criteria and were completely assessed (see Figure 1 for study selection and Table 1 for a summary of selected studies). Nine studies are concerned with cardiovascular diseases, 18 neurodegenerative disorders, and 20 cancer diseases. They had been conducted in the United States and Canada (*n* = 22), Europe (*n* = 22), Australia (*n* = 1), Israel (*n* = 1) and the islands of Flores and S.Miguel (*n* = 1). Included studies have been classified according to the disease for which patients were tested (Table 1 first column), while the study design is reported in Table 2.

**Table 2:** Moments of evaluation, before and after the genetic test, per study.

**Before genetic test**			**After genetic test**			
**Author**	**Year**	**Before disclosure of results**	** < or = 6 weeks after disclosure genetic test result**	**>6 weeks after disclosure genetic test result**	**1 year after disclosure genetic test result**	**>1 year after disclosure genetic test result**
Hickey et al.	2014		Not defined		
Hickey et al. (b)	2014		Not defined		
Christiaans et al.	2009				+
Jones & Clayton	2012	+				+ (18 months)
van Maarle et al.	2001	+	+		
Marteau et al.	2004	+	+	+ (6 months)	
Hietaranta-Luoma et al.	2015	+		+ (10 weeks and 6 months)	+
Hendriks et al.	2008	+	+			+ (18 months)
Legnani et al.	2006	+	+		
Decruyenaere et al.	2003	+			+	+ (5 years)
Licklederer et al.	2008		Not defined		
Almqvist et al.	2003	+	+	+	+	+ (2 and 5 years)
Timman et al.	2004	+	+	+		+ (1.5, 3, 7–10 years)
Larsson et al.	2006	+		+	+	+ (2 years)
Horowitz et al.	2001	+		+ (3 and 6months)	+
Gargiulo et al.	2009			+	+
Wahlin et al.	2000	+		+ (2 and 6 months)	+	+ (2 years)
Witjes-Ané et al.	2002	+				+ (18 months)
Surampalli et al.	2015	+			+
Gonzalez et al.	2012					+ (5 years)
Gooding et al.	2006					Not defined
Linnenbringer et al.	2010	+		+	
Cassidy et al.	2008		+		+
Green et al.	2009	+	+	+ (6 months)		+ (1 year)
Chao et al.	2008				+
Vernarelli et al.	2010		+		
Romero et al.	2005		+ (1 month and 4 months)		
Aspinwall et al.	2013	+	+	+	+	+ (2 years)
Lammens et al.	2010	–	Not defined		
Di Prospero et al.	2001		Not defined		
Ertmanski S et al.	2009	+	+		+
Hamilton et al.	2009				
Vos et al.	2012			+		+ (3- to 4-years)
Katapodi et al.	2011		Not defined		
Lodder et al.	2000	+	Not defined		
Meiser et al.	2002	+	+	+	+
Rini et al.	2009		+	+	+
Smith et al.	2008	+	+	+ (3 and 6 months)	
Dougall et al.	2009	+	+	+ (3 and 6 months)	
Samson et al.	2014			+ (6 months)	
Arver et al.	2004	+	+	+ (2 and 6 months)	+
Claes et al.	2004		Not defined		
Manchanda et al.	2014		Not defined		+	+(2, 3 years)
Shiloh et al.	2011				+(Up to 4 years after)	+
Claes et al.	2005	+			+
Andrews et al.	2004	+	+	+	+
Reichelt et al.	2004	+	+		

### Cardiovascular Disease

Genetic testing for cardiovascular diseases is usually performed to detect users' susceptibility to conditions that affect the heart muscle, inherited heart conditions that might cause arrhythmias or risk factors which could cause a heart attack. Some of these conditions may require changes in lifestyle or medical therapy. Studies taken into examination evaluated the impact of genetic testing related to different clinical conditions (Hickey et al., 2014a,b), such as Long qt syndrome (Hendriks et al., 2008), thrombophilia (Legnani et al., 2006), cardiomyopathy (Christiaans et al., 2009), arterial hypertension (Jones and Clayton, 2012), and familial hypercholesterolaemia (Van Maarle et al., 2001; Marteau et al., 2004). In general, these studies used similar scales for the assessment of the quality of life and perception of risk (SF-36, IPQ-R) and for evaluating the psychological impact and wellbeing (STAI, HADS, IES). Psychological aspects mainly concerned the anxiety-depressive symptoms and the subjective distress caused by the “traumatic event” of genetic risk communication.

Results showed that no negative impacts on quality of life and no serious increase in distress or anxiety levels were registered after receiving genetic test results (Van Maarle et al., 2001; Marteau et al., 2004; Legnani et al., 2006; Hickey et al., 2014a,b). Anxiety levels were overall moderate and tended to last over time only if the genetic test result was associated with uncertain physiological data (Hendriks et al., 2008) or in case of marked clinical conditions, such as in patients with symptoms of hypertrophic cardiomyopathy before DNA testing (Christiaans et al., 2009). Hietaranta-Luoma et al. (2015) evaluated the impact of genetic testing for the Apolipoprotein E (ApoE, a protein involved in Alzheimer's disease and cardiovascular disease and mapped to chromosome 19). They reported that in high-risk subjects the genetic information combined with personal health status influenced the levels of anxiety, and promoted the short-term reduction of risk factors for cardiovascular disease. One study investigated arterial hypertension (Jones and Clayton, 2012) and detected distress symptoms in patients before undergoing genetic testing, comparable to PTSD symptoms. These symptoms significantly decreased after the reception of genetic results, in both carriers and non-carriers.

Even the quality of life after genetic test results was influenced by patients' difficulties in managing mental states, compared to other aspects. Hickey et al. (2014b) found that mental difficulties, assessed by the SF-36, were higher (59.9 ± 5.3) if compared to physical components, which resulted within normal ranges (46.2 ± 6.6), whereas Christiaans et al. (2009) clarified that levels of distress and impact on mental components did not significantly differ from the average of the general population who never underwent a genetic test. Finally, Marteau et al. (2004) showed that genetic testing for patients, already aware of their risk, does not affect their sense of control over the condition (hypercholesterolemia) but influenced their beliefs on how effectively achieve control on their health (e.g., with drug assumption).

### Neurodegenerative Disorders

Genetic testing for neurodegenerative disorders are usually performed: (a) for diagnostic purposes, (b) to determine if a person, who has a family history of disease, is a mutation carrier and thus he/she is at risk to develop the disorder or could have an affected offspring. Currently, no therapies exist for complete remission of these pathologies. Studies we have collected primarily investigated risk related to Alzheimer disease (AD) and Huntington disease (HD). Two studies devoted attention to other neurodegenerative disorders, such as the Machado-Joseph disease (MJD)(Gonzalez et al., 2012) (which causes symptoms like spasticity, difficulty with speech and swallowing, weakness in arms and legs, frequent urination) and mutation to VCP gene (Surampalli et al., 2015) (which along with the inclusion body myopathy it causes frontotemporal dementia).

Huntington Disease (HD) is a dominantly transmitted neurodegenerative disorder: genetic analysis detects, with 100% of certainty, the presence of the mutation gene, confirming the status as a carrier of the condition (Evers-Kiebooms and Decruyenaere, 1998). The outcomes of gene testing can rarely fall within the reduced penetrance range (36–39 CAG repeats), whereby individuals may or may not develop symptoms of the disease; or individuals may be carriers of intermediate alleles (27–35 CAG repeats) and will not develop symptoms of the disease themselves, but their children will be at-risk of HD (Myers, 2004).

Tools used to evaluate the psychological impact of genetic testing for HD predominantly measured anxiety and depression (STAI, BDI), the traumatic impact of the event “genetic test results communication” (IES) or severe psychological symptoms, up to suicidal ideations (see Table 1).

Most authors highlighted the presence of depression and suicidal ideation in a significant percentage of participants, even before undergoing genetic testing (Robins Wahlin et al., 2000; Horowitz et al., 2001; Larsson et al., 2006; Gargiulo et al., 2009), with higher psychological suffering and negative impact on QoL for those with neurological symptoms (Horowitz et al., 2001). Licklederer et al. (2008) found that patients, with mutation and already manifesting HD symptoms, had higher levels of depression and lower levels in QoL indexes, compared to gene carriers without symptoms and non-carriers. Moreover, they showed that depression values in HD gene carriers were related to unfavorable genetic test result in conjunction with negative social and relational conditions (e.g., low perceived social support and being childless). Summarizing, higher levels of depression and lower quality of life were registered in patients with manifest HD or neurological impairments (Horowitz et al., 2001; Licklederer et al., 2008).

Interestingly, Gargiulo et al. (2009) found that 27% of non-carriers (asymptomatic) do not positively elaborate the favorable genetic results whereas Robins Wahlin et al. (2000) showed that non-carriers had a very high frequency of suicide ideations. Another study has also shown that non-carriers tended to develop avoidant or intrusive styles as a reaction to the stressful event (genetic test results) over time (Timman et al., 2004).

Considering the long-term impact of genetic tests for HD, several studies revealed the presence or the increase in depressive symptoms, suicidal ideations, hopelessness, and aggressive reactions in gene carriers (Robins Wahlin et al., 2000; Witjes-Ané et al., 2002; Almqvist et al., 2003; Timman et al., 2004; Larsson et al., 2006; Gargiulo et al., 2009), except for the study of Decruyenaere et al. (2003), showing a significant decrease of depressive symptoms after 1 year, both in gene carriers and non-carriers.

Concerning Alzheimer disease (AD), currently ApoE testing is used in clinical settings to identify people who may have an increased risk of developing AD, whereas other genetic tests investigate the presence of autosomal dominant mutations (in genes PSEN1, PSEN2, and APP which are more predictive for disease development (Goldman et al., 2011).

The REVEAL studies (Chao et al., 2008; Green et al., 2009; Vernarelli et al., 2010) showed that ApoE carriers were not more anxious, depressed, or test-related distressed than people who did not receive any information about their genotype (Green et al., 2009). The levels of anxiety, depression, and distress were below clinical thresholds both in carriers and non-carriers, with a significant distress reduction among those who learned that they were ApoE negative. People who were highly distressed before undergoing genetic testing were more vulnerable to emotional difficulties after outcome disclosure, but distress values were well below clinical thresholds for clinical concern (Green et al., 2009). Romero et al. (2005) described that a small percentage of ApoE gene carriers felt depressed (15–30%) or worried (11–22%). A small percentage also felt relieved.

The study by Cassidy et al. (2008) found that participants who received a positive result for a deterministic mutation experienced the same levels of distress experienced by those receiving positive results for genetic susceptibility testing (ApoE). The same study reported that after 1 year from result disclosure the majority of participants did not experience clinically significant distress.

Concerning more in detail long-term results or changes in health-related behaviors, 12 months after ApoE results, carriers reported changes in lifestyle (diet, physical exercise, and medication or vitamin intake) more often than non-carriers or the nondisclosure group (Chao et al., 2008). A positive correlation between genetic susceptibility testing for AD (an APOE epsilon4+ genotype status) and changes in vitamin intake was also confirmed by Vernarelli et al. (2010), despite there is no evidence that supplement use reduces the risk of AD.

Finally, Linnenbringer et al. (2010) showed that people who accurately recalled their AD disease risk assessment (the risk percentage) tended to perceive their risk higher than the percentage of risk they were given (below clinical thresholds).

Finally, Gooding et al. (2006) interviewed a group of people at high risk for AD (because of relatives affected by AD), and genetic testing was estimated valuable information to improve personal control on health and guide future decisions.

In rare pathologies such as Machado Joseph disease (Gonzalez et al., 2012), anxiety levels were from moderate to severe in half of the participants (52.6%). Five years later quality of life was significantly more compromised in symptomatic people, confirming an impact of the appearance of first symptoms on the psychological state. Meanwhile in VCP genetic testing (Surampalli et al., 2015) were found similar results in anxiety levels as for Alzheimer disease.

### Cancer

Genetic testing for cancer is usually performed in pre-symptomatic conditions (the user never developed any symptom related to the cancer disease), or after an episode of cancer diagnosis, to know if there is a hereditary cancer syndrome and/or a risk of relapse.

Most of the articles focused on the risk of developing ovarian and breast cancer, by examining the presence of BRCA1 and BRCA2 mutations (see Table 1). Three studies, respectively, investigated the impact of genetic mutations responsible for pancreatic cancer and melanoma (Aspinwall et al., 2013), colon cancer along with breast cancer (Arver et al., 2004), and Li-Fraumeni Syndrome (Lammens et al., 2010). Studies on BRCA testing used several tools (STAI, IES, SCL-90, BDI, HADS the most used ones) assessing anxiety, post-traumatic stress disorders, psychopathological symptoms and depression, and showed quite heterogeneous results.

Breast and ovarian cancer were overall perceived as having the same seriousness independently by genetic test results (Claes et al., 2004). Many authors revealed the poor influence of genetic tests on anxiety and distress (distress levels within normal ranges), without significant difference between gene carriers and non-carriers (Andrews et al., 2004; Claes et al., 2005; Ertmanski et al., 2009). These results suggest that genetic testing for BRCA does not cause adverse psychological reactions. Four studies reported slightly greater levels of anxiety and negative psychological outcomes in gene carriers (Lodder et al., 2001a; Meiser et al., 2002; Katapodi et al., 2011; Shiloh et al., 2013) whereas Vos et al. (2012) specified that these anxiety levels would be mediated by individual risk perception and concerns about their own relatives' heredity-likelihood. Gene carriers and probands showed to be more distressed and negatively influenced by genetic test results, even because they were concerned about their offspring and experienced decisional conflicts toward their relatives (Claes et al., 2004; Rini et al., 2009; Katapodi et al., 2011). Three studies (Reichelt et al., 2004; Ertmanski et al., 2009; Manchanda et al., 2015) investigated the experience of genetic testing and risk perception in people with a family history or personal history of illness, comparing them with healthy people or people without previous family experience of disease, and they found conflicting results. Manchanda et al. (2015) demonstrated that there were no differences in levels of anxiety and distress based on the presence/absence of a family history of disease, while Reichelt et al. (2004) and Ertmanski et al. (2009) reported higher levels of distress in people who have already had a diagnosis and/or cancer experience. Women with a personal cancer history tended to enact concrete coping strategies more than women without previous experience with cancer (Dougall et al., 2009).

Finally, there are studies which reported satisfaction and positive consequences of having carried out the genetic test for breast/ovarian cancer susceptibility and thus discovering something about the presence of a mutation. In particular, receiving a positive result increased the perception of risk (Di Prospero et al., 2001; Claes et al., 2005; Katapodi et al., 2011; Vos et al., 2012), which correlated with more frequent screenings and checkups, and with a sense of self-efficacy (Di Prospero et al., 2001; Hamilton et al., 2009; Shiloh et al., 2013).

Long-term results showed that levels of test-related distress decreased in the first 4/6 months (Andrews et al., 2004; Arver et al., 2004; Smith et al., 2008), then enduring at low levels after years (Andrews et al., 2004; Manchanda et al., 2015), with an impact on surveillance actions up to 4 years after test disclosure (Shiloh et al., 2013).

Concerning other cancers, in Aspinwall et al. (2013) gene carriers for pancreatic cancer and melanoma increased preventive screening for themselves and their families, thanks to informative genetic test results. A small percentage of patients reported clinically relevant levels of distress related to genetic testing for p53 germline mutation (Lammens et al., 2010). Distress was higher for patients with a lack of social support, as was the case for Huntington Disease (Licklederer et al., 2008).

## Discussion

In the last decades, clinical application of genetic testing for diagnosis and prevention has gained more importance to such an extent as to create a market where patients can obtain information on genetic risk in complete autonomy (Su, 2013; Oliveri et al., 2015, 2016b; Oliveri and Pravettoni, 2016). In this framework, there are many possible psychological reactions and related issues worthy of consideration, such as risk perception and perceived controllability after a positive result for a mutation, or concerns about transmitting susceptibility for a disease to future generations.

With this contribution, we aimed to sound out possible differences in psychological reactions to predictive genetic testing based on different disease categories. To date, there are no reports that compare the psychological impact of genetic testing for cardiovascular, neurodegenerative and cancer diseases.

Our review shows that there is no significant increase in distress levels or adverse impact on the quality of life in subjects who undergo a genetic test for cardiovascular diseases; when higher distress is present it does not exceed the clinically significant threshold (Van Maarle et al., 2001; Marteau et al., 2004; Legnani et al., 2006; Hickey et al., 2014b). The psychological distress is related to a full-blown clinical condition in addition to a positive genetic result (Hendriks et al., 2008; Christiaans et al., 2009). Overall people maintain confidence in being able to cope with their risk, even though they modify the opinion on how to address this risk: they tend to believe that lifestyle might be useless to face their “genetic predisposition,” and they need other “more concrete” methods of prevention, such as drug therapies (Marteau et al., 2004). In our opinion, these trends arise from a “deterministic” interpretation of genetic data, and the lack of evidence concerning the effects of lifestyle modifications in the disease course. Changes in lifestyle only concern people who already have physical symptoms (Marteau et al., 2004; Hietaranta-Luoma et al., 2015) and are at higher risk of adverse heart conditions, although these lifestyle changes have short duration (Hietaranta-Luoma et al., 2015). We hypothesize that people with full-blown symptoms are motivated to gather all possible health-related information, including genetic risk information, in order to manage their risk of developing the disease. In general, our review shows that genetic risk for cardiovascular disease is perceived to be manageable, and this might also be due to the existence of screenings to prevent it and possible treatments.

Concerning neurodegenerative disorders, studies put more attention on anxiety and depression symptoms, since these disorders usually have relevance on complex emotions such as embarrassment and social withdrawal (Levenson et al., 2014), affect family relations and put carriers at risk of social discrimination (Perry, 1981; Craufurd and Harris, 1986). Our review describes marked negative psychological impact after positive genetic results for Huntington Disease in patients who have depressive symptoms already before undergoing genetic testing, including suicidal ideation, which are increased also by the presence of adverse relational/family situations (Robins Wahlin et al., 2000; Horowitz et al., 2001; Larsson et al., 2006; Licklederer et al., 2008; Gargiulo et al., 2009). Differently from other chronic diseases, a negative genetic result for HD does not reassure, but it causes negative emotions. This reaction might be due to the uncertainty of results and a lack of “response” for the etiology of cognitive symptoms, when present, or sense of guilty toward family members that have the diseases (Robins Wahlin et al., 2000; Timman et al., 2004; Gargiulo et al., 2009). However, we believe that these results for Huntington disease should be taken with the due caution called by the fact that, often, the evidence was based on participants with previous psychiatric history (Robins Wahlin et al., 2000; Almqvist et al., 2003; Larsson et al., 2006; Gargiulo et al., 2009). Thus, we cannot rule out that, for example, a manifestation of suicidal ideation can be ascribable to this previous psychiatric history rather than to the positive or negative genetic result. Future studies should settle this issue.

Negative psychological impact of genetic testing in gene carriers for HD persists over time (Almqvist et al., 2003; Timman et al., 2004; Larsson et al., 2006), and it might be due to the regret for having undergone the test, anticipating life change limitations (Hagberg et al., 2011).

We argue that in addition to the regret for getting such genetic information, negative reactions may be understandable in light of the certainty these people have to develop HD in the future, the perception of something uncontrollable and fatal, alongside the complete absence of valid therapies and inevitable cognitive decline (Gooding et al., 2006). The decision in undergoing predictive genetic testing, in this case, could be a coping strategy (Gooding et al., 2006)acted to redirect important life decisions.

Slightly different seems to be the impact of genetic analysis for Alzheimer disease. Effects of genetic test results are comparable to those described for cardiovascular diseases, since distress anxiety and depression are below clinically significant thresholds, even for gene carriers, and these results concern both APOE and autosomal dominant mutation testing (Cassidy et al., 2008; Green et al., 2009; Linnenbringer et al., 2010). Therefore, the test is overall experienced as something useful to achieve a good degree of awareness and immediately act preventive behaviors to address the risk. Anyway, for Alzheimer's prevention behavioral changes are not always positive: for example, an increasing assumption of dietary supplements harmless, such as vitamin E, could give people a false perception of control on the health without any significant scientific evidence (Morris et al., 2002). For this reason, it would be beneficial to provide people with more information on how to effectively prevent AD, before providing the opportunity to undergo genetic testing.

Finally, in rare diseases, such as Machado Joseph, anxiety levels were prominent after genetic testing in at least half of patients studied (Gonzalez et al., 2012). These results are understandable in the light of an immediate impairment of daily life (e.g., spasticity, difficulty with speech and swallowing, weakness in arms and legs, frequent urination) and the fact that symptoms get worse over time.

Results on the impact of gene testing in cancer raise more complex and heterogeneous issues than in previous cases. From the emotional point of view, the levels of anxiety and depression decrease significantly after having received test results (Andrews et al., 2004; Arver et al., 2004; Reichelt et al., 2004; Claes et al., 2005; Smith et al., 2008; Ertmanski et al., 2009; Lammens et al., 2010; Aspinwall et al., 2013; Manchanda et al., 2015), and a positive effect emerges as regards screening behaviors as well (Hamilton et al., 2009; Shiloh et al., 2013). We must consider, in order to give an interpretation to the previous results, that breast and ovarian cancer are potentially preventable and early detection can guarantee to heal with effective treatments (Shaw and Bassi, 2001). If results are positive, screening or surgery could help patients reduce their risks, and immediate communication to family members about genetic risk can be crucial to prevent the “danger” of disease development. Deciding on how to address the risk means being able to “recommend” a pathway for prevention to their families (Lodder et al., 2001b; Katapodi et al., 2011; Vos et al., 2012). Preventive and prophylactic decisional pathways are no easy nor straight: risk-reducing prophylactic mastectomy on healthy breast goes along the risk of surgical side effects, body image modification, regrets in women who decided for this solution. Periodic screening is instead potentially accompanied by frequent negative thoughts and emotions (anxiety components).

People who have already had an experience of illness tend to actively cope with the risk of disease onset, although sometimes this is accompanied by higher levels of distress (Dougall et al., 2009). These levels of arousal, however, should not be necessarily perceived in a negative sense; on the contrary emotional arousal could be the engine for the decisional process in cancer care and for acting on coping strategies. Even for cancer, as already found for APOE and Alzheimer's, studies indicate that there are positive aspects reported by patients about having undergone genetic testing. These findings are related to an increase in screening behaviors and an increased sense of self-efficacy in managing the risk (Hamilton et al., 2009; Aspinwall et al., 2013; Shiloh et al., 2013). Summarizing, in cancer, if people get important information on time they can manage their risky or healthy behaviors enhancing the perception of control over their lives and direct it as they wish (e.g., surgery *vs*. screening).

This is not completely true for cardiovascular disease and Huntington disease, because cardiovascular disease genetic information and evidence about preventive medicine efficacy are completely uncertain and say “something less” about predisposition and prevention options; for HD, deterministic implications of genetic testing give a piece of information that is likely to be “too much information,” and thus perceived as uncontrollable.

Before to conclude, it is important to point out some limitations of the reviewed studies. The first limitation is that gender was not well balanced in all the selected studies and was not investigated as a factor that could influence the psycho-behavioral impact of genetic testing. We exclude papers on breast cancer (which investigated women samples), Li-Fraumeni Syndrome (LFS) (where men and female were equally balanced and female gender was associated with heightened levels of LFS-related distress), and on the risk for developing diseases which disproportionately affect women (e.g., the primary pulmonary arterial hypertension)(Jones and Clayton, 2012). In many studies about neurodegenerative diseases, women were overrepresented (Cassidy et al., 2008; Green et al., 2009; Vernarelli et al., 2010; Gonzalez et al., 2012) and we cannot exclude that the results could be affected by general gender bias. Chao et al. (2008), for example, claimed that the REVEAL study participants were mainly women. Therefore the results may not be generalized to all population who might qualify for APOE genotype testing in the future.

In most studies concerning cardiovascular disease, males and female samples seemed to be well balanced, except for Hietaranta-Luoma et al. (2015) who evaluated more females. Nevertheless, gender differences, when investigated (Legnani et al., 2006; Jones and Clayton, 2012; Hickey et al., 2014a) did not relate to any of the measures of patient well-being.

A second limit was that not all the studies considered participants' educational level as a factor that could correlate with the decision to undergo a genetic test and its psycho-behavioral impact. In the selected studies participants had at least high school education (10–12 years of education completed)(Lodder et al., 2001b; Van Maarle et al., 2001; Witjes-Ané et al., 2002; Almqvist et al., 2003; Claes et al., 2004; Gooding et al., 2006; Legnani et al., 2006; Licklederer et al., 2008; Smith et al., 2008; Christiaans et al., 2009; Rini et al., 2009; Vernarelli et al., 2010; Gonzalez et al., 2012; Vos et al., 2012; Hickey et al., 2014b) or were predominantly highly educated (Andrews et al., 2004; Claes et al., 2005; Dougall et al., 2009; Vernarelli et al., 2010; Aspinwall et al., 2013; Shiloh et al., 2013; Manchanda et al., 2015). Educational level was associated to a better recall of disease risk information (Linnenbringer et al., 2010), to a higher response rates in the follow up (Almqvist et al., 2003), or was not an influential predictor of the psycho-behavioral measures (Robins Wahlin et al., 2000; Meiser et al., 2002; Andrews et al., 2004; Claes et al., 2005; Legnani et al., 2006; Licklederer et al., 2008; Smith et al., 2008; Green et al., 2009; Rini et al., 2009; Jones and Clayton, 2012; Shiloh et al., 2013). Other studies included in our review did not perform analysis based on the educational level, and future studies should address this issue.

Finally, some of the results presented in these studies are based on small sample sizes (Robins Wahlin et al., 2000; Di Prospero et al., 2001; Andrews et al., 2004; Hamilton et al., 2009; Gonzalez et al., 2012; Hickey et al., 2014b; Samson et al., 2014; Surampalli et al., 2015) and are not cross-cultural. Therefore, it is possible that ethnicity and cultural aspects may play a role in determining the psychological implications of genetic testing.

## Conclusions

This review presented a comprehensive overview of the psychological impact of genetic testing across the most common chronic adults' diseases. The information level of genetic data varies according to the type of test. Along with this aspect, each of us has a specific perception of disease categories, for which genetic testing is available. Risk perception, worry, and other psychological reactions depend, for instance, on the perceived controllability and existing therapies to manage the illness; it is essential to proceed with an assessment of such factors along with the provision of genetic information.

Over the last 20 years we have witnessed a proliferation of investments in genomics research in order to study disease prevention, disease treatments, better drug therapies, and genetic paths to cure, and, thanks to media coverage such as Angelina Jolie's case (Evans et al., 2014), the psychological impact of these discoveries has gradually become more and more important.

For these reasons, in the present review, we tried to understand better how genetic testing users' perceptions of developing specific diseases affect their psychological well-being and lifestyle. Understand psycho-behavioral reactions could be an important starting point for an effective clinical application of genetic testing and to organize personalized care plans, which can drive patients to self-determination of a healthy lifestyle and to make appropriate decisions for their health.

## Author Contributions

SO and FF contributed to the design and implementation of the research, to the analysis of the results and the writing of the manuscript. AM contributed to the writing of the manuscript, and GP supervised all the process, was in charge of overall direction and planning.

### Conflict of Interest Statement

The authors declare that the research was conducted in the absence of any commercial or financial relationships that could be construed as a potential conflict of interest.
